# Genome Sequence of *Jannaschia aquimarina* GSW-M26, a Member of the *Roseobacter* Clade

**DOI:** 10.1128/genomeA.00353-15

**Published:** 2015-04-23

**Authors:** Sonja Voget, Stefani Maria Díaz Valerio, Avril Jean Elisabeth von Hoyningen-Huene, Praveen Kumar Nattramilarasu, Katharina Vollheyde, Shengbin Xiao, Rolf Daniel

**Affiliations:** a Genomic and Applied Microbiology and Göttingen Genomics Laboratory, Georg-August-University, Göttingen, Germany; b Members of the Applied Bioinformatics in Microbiology Course of the Microbiology and Biochemistry MSc/PhD program, Georg-August-University, Göttingen, Germany

## Abstract

The Gram-negative alphaproteobacterium *Jannaschia aquimarina* GSW-M26 (DSM 28248) is a member of the *Roseobacter* clade. The size of the draft genome is 4.1 Mb. Genome analysis revealed the presence of genes encoding a complete gene transfer agent and aerobic anoxygenic photosynthesis. The latter indicated a photoheterotrophic lifestyle.

## GENOME ANNOUNCEMENT

The Gram-negative alphaproteobacterium *Jannaschia aquimarina* GSW-M26 is closely related to *Jannaschia seosinensis* and *Jannaschia helgolandensis* (1) and belongs to the *Roseobacter* clade (2). Strain GSW-M26 (DSM 28248) is the type strain of the species and was isolated from seawater off the southern coast of Korea (1). A study of aerobic bacteriochlorophyll *a*-producing bacteria in the Baltic Sea showed that members of the genus *Jannaschia* are of high environmental significance as *Jannaschia*-related clones constituted up to 25% of all clones with the ability to perform aerobic anoxygenic photosynthesis (3). Despite this importance, only one genome sequence of *Jannaschia* species is available to date (4). *J. aquimarina* GSW-M26 (DSM 28248) was derived from the DSMZ (Braunschweig, Germany). The strain was grown in marine broth (MB 2216; BD Difco, Sparks, MD, USA) at 30°C. Chromosomal DNA was isolated with the MasterPure complete DNA purification kit (Epicentre, Madison, WI, USA). Preparation of paired-end sequencing libraries with the Nextera XT library preparation kit and sequencing of the libraries using the Genome Analyzer IIx were performed as described by the manufacturer (Illumina, San Diego, CA, USA). A total of 8,995,698 paired-end reads were derived from sequencing and trimmed using Trimmomatic version 0.32 (5). *De novo* assembly of all remaining reads with SPAdes version 3.5.0 (6) resulted in 81 contigs and 143.84-fold coverage. The draft genome sequence of *J. aquimarina* comprises 4.1 Mb and a GC content of 66.4%. Genome annotation was performed with Prokka version 1.10 (7). The draft genome harbored 1 rRNA operon, 51 tRNA genes, and 4,105 protein-encoding genes, of which 3,234 have a predicted function. The presence of a plasmid in *J. aquimarina* is indicated by genes encoding a plasmid replication system of the RepABC type. This differs from *Jannaschia* sp. CCS1, which carries a plasmid with a RepA-type plasmid replication system (8). The complete cluster for a gene transfer agent was identified in the *J. aquimarina* genome. Gene transfer agent–mediated horizontal gene transfer and the corresponding systems have only been found in *Alphaproteobacteria* but not in all members of the *Roseobacter* clade (9). It was suggested that these systems represent a potential adaptation mechanism of marine bacteria to maintain their flexibility in dynamic marine environments (10). In addition, a photoheterotrophic lifestyle of *J. aquimarina* is indicated by a gene cluster typical for aerobic anoxygenic photosynthesis. Genes encoding type I and II carbon monoxide dehydrogenases are present in *Jannaschia* sp. CCS1 (4), but type I dehydrogenase, which is essential for carbon monoxide oxidation (11), is absent in *J. aquimarina*. Interestingly, genes similar to *sox* genes that mediate the oxidation of sulfide or thiosulfate were not observed in the genome of *J. aquimarina*, although they were identified in the majority of the genomes derived from members of the *Roseobacter* clade (2). These findings indicate that *J. aquimarina* GSW-M26 performs a photoheterotrophic but not a chemolithotrophic lifestyle.

### Nucleotide sequence accession numbers. 

This whole-genome shotgun project has been deposited at DDBJ/EMBL/GenBank under the accession number JYFE00000000. The version described in this paper is version JYFE01000000.
